# Exploring the link between home garden use and severe obesity: Insights from a nationwide survey in Tuvalu

**DOI:** 10.7189/jogh.13.04097

**Published:** 2023-09-01

**Authors:** Po-Jen Lin, Tai-Lin I Lee, Maria Soledad Hershey, Chih-Wei Shih, Selotia Tausi, Vine Sosene, Pauke P Maani, Malo Tupulaga, Stephanie M Wu, José Francisco López-Gil, Yuan-Hung Lo, Shi-Chian Shiau, Yu-Tien Hsu, Chia-Rui Chang, Chih-Fu Wei

**Affiliations:** 1 Taiwan International Cooperation and Development Fund (ICDF), Taipei, Taiwan; 2 Department of Medicine, Nuvance Health Danbury Hospital, Danbury, Connecticut, USA; 3 Johns Hopkins University Bloomberg School of Public Health, Baltimore, Maryland, USA; 4 Division of Medical Imaging, Department of Radiology, Far Eastern Memorial Hospital, New Taipei city, Taiwan; 5 Department of Environmental Health, Harvard T.H. Chan School of Public Health, Boston, Massachusetts, USA; 6 Taiwan Technical Mission to Tuvalu, Funafuti, Tuvalu; 7 Department of Agriculture, Tuvalu Ministry of Local Government and Agriculture, Funafuti, Tuvalu; 8 Department of Public Health, Tuvalu Ministry of Health, Funafuti, Tuvalu; 9 Department of Biostatistics, Harvard University T.H. Chan School of Public Health, Boston, Massachusetts, USA; 10 Navarrabiomed, Hospital Universitario de Navarra, Universidad Pública de Navarra, IdiSNA, Pamplona, Navarra, Spain; 11 One Health Research Group, Universidad de Las Américas, Quito, Ecuador; 12 Department of Social & Behavioral Sciences, Harvard T.H. Chan School of Public Health, Boston, Massachusetts, USA

## Abstract

**Background:**

Obesity is prevalent and increasing but understudied across Pacific Islanders. Tuvalu is a South Pacific country with a high obesity rate and faces multiple threats of food insecurity. Home garden serves as a sustainable food source and can be a possible intervention for the obesity pandemic in Tuvalu. This study investigated Tuvaluans’ home garden use and obesity, and explored factors associated with increased use of home gardens.

**Methods:**

We conducted a nationwide, cross-sectional study in Tuvalu during 2022. Structured questionnaires were administered during the in-person interviews, and trained interviewers measured the height and weight of each participant. The association between home garden use, obesity and severe obesity were tested with univariate and multivariable logistic regression. We also applied overlapping weights to balance the distribution of baseline demographic factors.

**Results:**

The average body mass index was 34.87 kilogrammes (kg) / square metre (m^2^) among the study population of 1024 adults (630 from Funafuti and 394 from other islands in Tuvalu). Overall, people having home gardens was associated lower odds for severe obesity compared to those without a home garden in overlap weighting models (odds ratio (OR) = 0.946, 95% CI = 0.897-0.997, *P* = 0.039) and the association was stronger in Funafuti (OR = 0.927, 95% CI = 0.866-0.991, *P* = 0.027) than in the outlying islands (OR = 0.967, 95% CI = 0.889-1.052, *P* = 0.435). Furthermore, increased age was positively associated with having a home garden in Funafuti, and smoking showed an inverse association.

**Conclusions:**

Having a home garden is associated with lower odds of severe obesity in Tuvalu, and the association is stronger in Funafuti. Smokers are less likely to have home gardens, and increased age is positively associated with having home gardens. These findings promote more home garden utilisation and provide evidence for targeted interventions in Tuvalu.

Obesity has become a global crisis. In 2016, it was estimated that 13% of adults were obese worldwide [1]. Obesity is a risk factor for a number of non-communicable diseases (NCD) such as metabolic syndrome, cardiovascular diseases, osteoarthritis, and some types of cancers (e.g., breast, colon) [2]. These comorbidities can cause a considerable burden on the vulnerable health economic system globally, especially the Pacific Islands which have overwhelming obesity rates [3].

Tuvalu, a South Pacific Island country, has a high prevalence of obesity (59.9% in adult women and 51.5% in adult men) [4]. The driving forces of obesity in Tuvalu come from individual factors (attitude and behaviours), structural factors (such as income) and food insecurity that prompt the consumption of high-calorie imported food. Although strategies like dietary modification and exercise are encouraged to prevent obesity, they are difficult to be habitualised [5].

Home gardens seem to alleviate the obesity pandemic in multiple dimensions [6-9]. They produce benefits on participants’ physical, mental, and social well-being [6,10-12]. Home garden use can provide affordable fresh fruits or vegetables to the local people and increase physical activity [12-14]. In addition, past research has identified home gardens as sustainable sources of healthy and affordable food products and exert a positive impact on health awareness and community-building [11,15]. For the Pacific Island countries specifically, home gardens have the potential to promote agricultural diversification and maximise self-sufficiency, as described in the national development plans for Fiji, Kiribati, Papua New Guinea, Tonga, Vanuatu, and Western Samoa [8]. However, no study discusses the association between obesity and home garden use in this region.

Home garden interventions in Tuvalu were initiated and implemented by the collaboration of Tuvalu Department of Agriculture and Taiwan International Cooperation and Development Fund (Taiwan ICDF) over the past decade [16]. These institutions promoted home garden use by providing free seedlings and technical support to transform free land into fertile home gardens. Therefore, the aim of the present study was 2-fold: first, to investigate the association between home garden use and obesity in Tuvalu; and second, to determine the factors that predict the use of home gardens.

## METHODS

### Study population and health outcomes

We carried out this study in Funafuti, the main island as well as eight outlying islands in Tuvalu. These outlying islands can be categorised into three groups by their relative geographical locations: 1. North islands (including Nanumea, Nanumaga, and Niutao), 2. Middle islands (including Vaitupu, Nukufetau, and Nui) and 3. South islands (including Nukulaelae and Niulakita).

Each of the island groups connects with Funafuti with scheduled passenger ships and we showed the number of participants on each island in Table S1 in the **Online Supplementary Document**.

Face-to-face interviews were carried out in household settings by pre-trained, local interviewers. Convenience sampling was chosen due to the absence of personal identification number among Tuvaluans, coupled with the lack of address or road names in the country. Also, people in Tuvalu usually gathered in the community and the travel restrictions during COVID-19 and our local staff can increase participant recruitment through convenient sampling [17].

In Funafuti, interviewers visited households in the seven villages on Fongafale islet (Fakaifou, Senala, Alapi, Vaiaku, Lofeagai, Teone, and Tekavatoetoe). In the outlying islands, interviewers took the passenger ships, and visited households around the island wharf, which are the main residential area. Through convenience sampling, we selected one or two study participants (aged >18) from each household. Informed consent was obtained from each interviewee, and only Tuvaluan citizens (including native Tuvaluans and immigrants) were approached.

The interview questionnaire was structured to collect information regarding sociodemographics, health behaviours and attitude, home garden use, and biometrics information. Our study used a body mass index (BMI) to assess the interviewee’s nutritional status. The local research team staff recorded height with tape measure and weight with electronic weigher during interviews. We defined BMI>30 kilogrammes (kg) / square metre (m^2^) as obesity, and BMI>40 kg / m^2^ as severe obesity following the World Health Organization criteria [18].

The study protocol was approved by Tuvalu department of health. The whole data collection process was done between February and May 2022.

### Outcomes of interest

The outcomes of interest of this study are obese and severely obese, defined by participants’ body mass index (BMI). We defined BMI 30-39 as obese, and BMI>40 as severely obese following the World Health Organization criteria [18]. The local research team staff recorded height with tape measure and weight with electronic weigher during interviews. The research team then calculated an individual’s BMI based on the measured weight and height data.

### Exposure of interest

The exposure of interest of this study is home garden use. We focus on the utilisation of home gardens in this study because people in Tuvalu traditionally obtained food near their households. In addition, the Tuvalu government has been collaborating with the Taiwan ICDF on promoting climate-resilient garden use to increase the yield of fresh vegetables and fruits. We asked participants about their home garden use status through a single question during the interview, “Does your family own a home garden?” This question is also translated in the Tuvaluan version as “E isi se otou fatoaga ite fale (Ao or Ikai)?” The responses were coded as a binary variable (yes or no).

### Covariate

Trained interviewers collected the covariates information during the survey. We determined the covariates based on background knowledge on home garden use and food consumption in Tuvalu, which included age, gender (female, male), education level (high school and above), income above the median (Australian dollar (AU$) 200, or US dollar (US$) 131.70 in this study), smoking, and medical history of metabolic diseases (based on self-reported history of hypertension, diabetes, or dyslipidaemia).

### Statistical analysis

Baseline characteristics of each study group were summarised using descriptive statistics (**Table 1**). To investigate the potential differences between residents of main island and outlying islands, we further stratified **Table 1** by residence status (**Table 2**). For the primary analysis, we assessed the marginal association between home garden use and obesity / severe obesity through univariate logistic regression. To account for potential confounding, we adjusted for gender, age, education level, NCD diagnosis, income, and smoking by applying both multivariate logistic regression and overlap weighting (**Table 3**). We applied multivariate logistic regression to explore variables potentially associated with home garden use (**Table 4**). We considered two-sided *P*-values less than 0.05 as statistically significant.

**Table 1:** Demographic characteristics distribution of the study population, stratified by with home garden use, in n (%) or mean (standard deviation (SD))

	Total study population (n = 1024)	No home garden (n = 705)	Home garden users (n = 319)	*P*-value
Age in years, mean (SD)	41.6 (16.1)	40.6 (15.4)	43.6 (17.3)	0.006
Female, n (%)	549 (53.6)	372 (52.8)	177 (55.5)	0.459
Income in AU$, mean (SD)	183.9 (477.2)	200.6 (534.8)	147.2 (312.3)	0.099
Education at college or above (%)	159 (15.6)	116 (16.5)	43 (13.5)	0.254
Living in outlying islands (%)	394 (38.5)	238 (33.8)	156 (48.9)	<0.001
BMI, mean (SD)	34.87 (8.08)	35.04 (8.14)	34.51 (7.94)	0.338
Non-communicable disease diagnosis (%)	232 (22.7)	156 (22.2)	76 (23.8)	0.619
Smoking (%)				0.535
*Daily*	281 (27.5)	200 (28.4)	81 (25.4)	
*Occasional*	68 (6.6)	48 (6.8)	20 (6.3)	
*Never*	674 (65.9)	456 (64.8)	218 (68.3)	

SD – standard deviation, AU$ – Australian dollar, BMI – body mass index

**Table 2:** Demographic characteristics distribution of the study population, stratified by island of residence and home garden use

	Funafuti (n = 630)			Outlying islands (n = 394)		
	**No home garden (n = 467)**	**Home garden users (n = 163)**	***P*-value**	**No home garden (n = 238)**	**Home garden users (n = 156)**	***P*-value**
**Baseline characteristics**						
Age in years, mean SD)	38.1 (14.6)	40.9 (16.7)	0.042	45.6 (15.8)	46.5 (17.5)	0.623
Female, n (%)	251 (53.7)	93 (57.1)	0.523	121 (50.8)	84 (53.8)	0.631
Income in AU$, mean (SD)	248.3 (636.1)	167.1 (376.4)	0.127	108.3 (210.0)	126.7 (227.6)	0.410
Education at college or above (%)	99 (21.3)	30 (18.4)	0.502	17 (7.1)	13 (8.3)	0.809
BMI, mean (SD)	35.10 (8.48)	33.95 (7.77)	0.128	34.91 (7.44)	35.10 (8.10)	0.807
Non-communicable disease diagnosis (%)	81 (17.4)	29 (17.8)	1.000	75 (31.5)	47 (30.1)	0.858
Smoking (%)			0.066			0.776
*Daily*	128 (27.5)	30 (18.4)		72 (30.3)	51 (32.7)	
*Occasional*	29 (6.2)	10 (6.1)		19 (8.0)	10 (6.4)	
*Never*	309 (66.3)	123 (75.5)		147 (61.8)	95 (60.9)	

SD – standard deviation, AU$ – Australian dollar, BMI – body mass index

**Table 3:** Multivariable regressions between home garden use on obesity and severe obesity prevalence, shown in odds ratio, 95% confidence interval and *P*-value*

	Unadjusted model	Fully adjusted model†	Overlap weight model‡
**Obesity**			
Overall	0.926 (0.689-1.246, *P* = 0.612)	0.888 (0.653-1.207, *P* = 0.448)	0.972 (0.917-1.031, *P* = 0.353)
Funafuti	0.847 (0.575-1.249, *P* = 0.402)	0.796 (0.530-1.194, *P* = 0.270)	0.951 (0.876-1.032, *P* = 0.231)
Other islands	0.955 (0.596-1.533, *P* = 0.850)	0.949 (0.583-1.544, *P* = 0.833)	0.988 (0.907-1.076, *P* = 0.779)
**Severe obesity**			
Overall	0.737 (0.529-1.027, *P* = 0.071)	0.725 (0.516-1.018, *P* = 0.063)	0.946 (0.897-0.997, *P* = 0.039)
Funafuti	0.610 (0.378-0.983, *P* = 0.042)	0.612 (0.375-1.000, *P* = 0.050)	0.927 (0.866-0.991, *P* = 0.027)
Other islands	0.846 (0.523-1.367, *P* = 0.494)	0.851 (0.519-1.394, *P* = 0.521)	0.967 (0.889-1.052, *P* = 0.435)

*Obesity was defined as body mass index (BMI)>30 kilogrammes (kg) / square metres (m^2^), and severe obesity as BMI>40 kg / m^2^.

†Adjusted for gender (male or female), age (grouped in ten years), education level (high school or above), non-communicable disease (NCD) diagnosis (having hypertension, diabetes or dyslipidaemia), income (>200 Australian dollar (AU$) or not), and smoking. Frequent consumers are people with response as “almost daily” or “several times a week” for each food item.

‡Weighted for gender (male or female), age (grouped in ten years), education level (high school or above), NCD diagnosis (having hypertension, diabetes or dyslipidaemia), income (>200 AU$ or not), and smoking.

**Table 4:** Multivariable regressions on possible predictors of home garden use, shown in odds ratio, 95% confidence interval and *P*-value*

Predictor variables for home garden ownership	Univariate model	Multivariable model
**Funafuti**		
Smoker	0.640 (0.423-0.953, *P* = 0.031)	0.649 (0.422-0.983, *P* = 0.045)
Aged 30-40 y	0.908 (0.547-1.484, *P* = 0.704)	0.927 (0.555-1.526, *P* = 0.767)
Aged 40-50 y	0.868 (0.454-1.591, *P* = 0.657)	0.847 (0.435-1.584, *P* = 0.613)
Aged 50-60 y	1.377 (0.804-2.325, *P* = 0.236)	1.427 (0.789-2.551, *P* = 0.234)
Aged 60-70 y	2.003 (1.069-3.700, *P* = 0.028)	2.031 (1.015-4.017, *P* = 0.043)
Aged >70 y	2.003 (0.710-5.315, *P* = 0.169)	2.304 (0.782-6.447, *P* = 0.116)
Female	1.143 (0.799-1.641, *P* = 0.465)	1.100 (0.752-1.615, *P* = 0.624)
Higher education	0.834 (0.523-1.300, *P* = 0.433)	0.869 (0.524-1.413, *P* = 0.579)
With NCD diagnosis	1.026 (0.635-1.622, *P* = 0.914)	0.693 (0.398-1.179, *P* = 0.184)
Higher income (>200 AU$)	0.807 (0.546-1.183, *P* = 0.278)	0.975 (0.633-1.491, *P* = 0.906)
**Other islands**		
Smoker	1.037 (0.684-1.569, *P* = 0.863)	1.119 (0.716-1.747, *P* = 0.621)
Aged 30-40 y	0.816 (0.441-1.501, *P* = 0.513)	0.819 (0.436-1.532, *P* = 0.532)
Aged 40-50 y	0.540 (0.255-1.106, *P* = 0.098)	0.568 (0.262-1.194, *P* = 0.142)
Aged 50-60 y	1.004 (0.542-1.855, *P* = 0.991)	1.070 (0.547-2.091, *P* = 0.842)
Aged 60-70 y	0.925 (0.477-1.782, *P* = 0.815)	1.013 (0.484-2.112, *P* = 0.972)
Aged >70 y	1.368 (0.582-3.226, *P* = 0.470)	1.484 (0.602-3.671, *P* = 0.389)
Female	1.128 (0.753-1.692, *P* = 0.559)	1.151 (0.743-1.786, *P* = 0.529)
Higher education	1.182 (0.547-2.498, *P* = 0.663)	1.251 (0.551-2.791, *P* = 0.586)
With NCD diagnosis	0.937 (0.603-1.449, *P* = 0.771)	0.909 (0.544-1.511, *P* = 0.713)
Higher income (>200 AU$)	0.947 (0.611-1.462, *P* = 0.808)	1.027 (0.617-1.703, *P* = 0.917)

y – year, NCD – non-communicable disease, AU$ – Australian dollar

*Adjusted for gender, age (grouped in ten years), education level, NCD diagnosis (having physician-diagnosed hypertension, diabetes, or dyslipidaemia), income, and smoking.

We chose the overlap weighting (OW) approach, a propensity score (PS) method to adjust potential confounding by weighing each individual with the probability of that individual being assigned to the opposite group [19]. Compared to the standard inverse probability weighting (IPW) method, which might suffer from extreme weights and lead to biased treatment effect estimates, the OW method has been demonstrated, both theoretically and in practice, to achieve exact balance and better precision [20,21]. Additionally, since OW mitigates the influence of individuals at the tail of the PS distribution, analysts do not have to exclude study participants with extreme weights, which could occur in IPW. Given the advantages mentioned above, we applied the overlap weighting approach in the current study.

### Sensitivity analysis

We used the standardised mean differences to examine the covariate balance after overlap weighting (Table S1 and Table S2 in the **Online Supplementary Document**). To account for potential change in behaviour after diagnostics of metabolic diseases, we excluded study participants with hypertension, diabetes, and dyslipidaemia to minimise this potential reverse causality. Table S2 in the **Online Supplementary Document** showed the association between home garden use, obesity, and severe obesity in the study population without NCDs. Table S3 in the **Online Supplementary Document** showed the association between home garden use, obesity, and severe obesity in the study population without NCDs in Funafuti. All analyses were conducted with R (version 4.0.4) [22].

## RESULTS

### Descriptive characteristics of the study population

We included 1024 adults in the analysis, with 549 (53.6%) females, 319 (31.2%) home garden users, mean age of 41.6 years old, and mean BMI of 34.87 kg / m^2^. Home garden users were older than non-users (43.6 years old for users, and 40.6 for non-users, *P* = 0.006). Outlying island residents were more likely to own home gardens compared to main island residents (48.9% vs. 33.8%, *P* < 0.001). No significant differences in gender, income, and education level were found between the users and non-users. Meanwhile, there were no significant differences in BMI, NCD diagnosis, and smoking status between the home garden users and non-users. The results were shown in (**Table 1**).

### Differences in baseline characteristics between residents of Funafuti and the outlying islands

We further stratified **Table 1** by residence status to account for potential differences in demographic characteristics between the main and outlying islands. No significant differences in gender, income, education levels, BMI, and NCD diagnosis were found between residents of the main and outlying islands. Among the 630 Funafuti residents, home garden users were older than non-users (40.9 years vs. 38.1 years, *P* = 0.042); Furthermore, there were fewer daily smokers among the home garden users, but the difference was not statistically significant (18.4% vs. 27.5%, *P* = 0.066). Among the 394 residents in the outlying islands, we did not identify significant demographic differences between home garden users and non-users. The results were shown in (**Table 2**).

### Association between home garden use and obesity / severe obesity in Tuvalu

There was no significant association between home garden use and obesity, either before or after adjusting for potential confounders (marginal odds ratio (mOR) = 0.926, 95% CI = 0.689-1.246, *P* = 0.612; adjusted odds ratio (aOR) = 0.888, 95% CI = 0.653-1.207, *P* = 0.448; aOR in the weighted model = 0.972, 95% CI = 0.917-1.031, *P* = 0.353). Meanwhile, the odds of severe obesity for home garden users was 0.946 times than the odds of severe obesity for non-users (aOR in the weighted model = 0.946, 95% CI = 0.897-0.997, *P* = 0.039). However, the confidence interval for both the unadjusted and adjusted models included the null.

We further examined the association by residence status. There was no association between home garden use and obesity, either among Funafuti residents or residents of outlying islands. At the same time, we observed home garden use was associated with a decrease in the odds of severe obesity among Funafuti residents (mOR = 0.610, 95% CI = 0.378-0.983, *P* = 0.042; aOR = 0.612, 95% CI = 0.375-1.000, *P* = 0.050; aOR in the weighted model = 0.927, 95% CI = 0.866-0.991, *P* = 0.027) but not among people living in outlying islands. These results were summarised in **Table 3**.

### Variables associated with home garden use

Among residents in Funafuti, current smokers were less likely to own a home garden compared to non-smokers (unadjusted (uOR) = 0.640, 95% CI = 0.423-0.953, *P* = 0.031; aOR = 0.649, 95% CI = 0.422-0.983, *P* = 0.045). Furthermore, people aged 60-70 years were more likely to own a home garden than those <30 years old (uOR = 2.003, 95% CI = 1.069-3.700, *P* = 0.028; aOR = 2.031, 95% CI = 1.015-4.017, *P* = 0.043). We did not identify any other associations for Funafuti. Among residents in the outlying islands, no associations were found between home garden use and gender, education, income, NCD diagnosis and other demographic factors. The results were shown in (**Table 4**).

### Sensitivity analysis

Similar to the primary analysis, we did not observe a strong association between home garden use and obesity either for those without an NCD diagnosis or for those living in Funafuti. At the same time, we did not observe an association between home garden use and severe obesity for those without an NCD diagnosis. Yet, the association between home garden use and severe obesity remained observable for the Funafuti residents (mOR = 0.552, 95% CI = 0.305-0.951, *P* = 0.039; aOR = 0.523, 95% CI = 0.284-0.916, *P* = 0.029; aOR in the weighted model = 0.919, 95% CI = 0.856-0.986, *P* = 0.019). The results were shown in Table S2 and Table S3 in the **Online Supplementary Document**.

## DISCUSSION

Overall, our findings indicate that home garden use in Funafuti was associated with lower odds of severe obesity from the overlap weighting model, with older age and non-smokers demonstrating higher home gardens utilisation. In contrast, we did not identify an association between home garden use and obesity in other outlying islands. Considering the greater effect of early obesity intervention over later stages [23], efforts should be directed towards encouraging home gardening among young people in Funafuti. The correlation between home garden use and non-smokers also highlights the interconnectedness of health concepts, suggesting that different dimensions of health concepts can synergistically impact behaviours [24].

The benefits of home gardens observed in our research are in line with previous studies in the UK that suggested domestic gardens as an important medium to community well-being [25]. Home gardening promotes health through multiple pathways. First, home gardens decrease the dependence on high-calorie and low-nutrition imported foods, which may explain in part the association between home garden use and decreased odds of severe obesity in Funafuti. Second, home gardens facilitate self-sufficiency and restore sustainability. Strengthening food production at the household level helps to attenuate the negative impact of COVID-19 pandemic to the global food supply chain [26]. One focus group study in Fiji and Saint Vincent and the Grenadines [15] regarded home gardens as a reliable anchor to address uncertainties in the imported food supply, which is especially important in the post COVID-19 era. Third, home gardens have strong links with local cultures and traditions. People are allowed to design their own gardens following the social context and decide which crops to raise [15]. Active participation in these processes gauge frequent reflections and self-awareness of food, which transcends the sole impact of food availability into mindfulness of healthier food choices [15]. Mindfulness of living healthy and adoption of health-promoting behaviours may be gauged by home garden participation as it has been negatively associated with being a smoker in Funafuti [27].

In this study, we found that home gardens were more prevalent among residents in outlying islands than in Funafuti, but the majority of the population (68.8%) in our study did not have home gardens. These results suggested that there are abundant opportunities for home garden promotion in Tuvalu. During our interview, we observed that Chinese cabbage and cucumber were the most common crops in home gardens, followed by tomatoes and green pepper. These vegetables fit not only into the climate conditions in Tuvalu but also the local dietary traditions, where meals are usually served with fresh-cut vegetables without complicated processing. These crops' low-maintenance and readily available features make them more popular in Tuvalu. In the Fruit and Vegetable Production and Nutrition Enhancement Project, the Tuvalu department of agriculture and Taiwan ICDF introduced a circular economy model which transforms waste materials into organic compost that strengthens agricultural activities. They hosted intensive training workshops and home gardening competitions in order to enhance local participation. Cooking demonstrations were also delivered to help people understand how to process certain kinds of vegetables that could promote willingness to establish home gardens and promote food security in the country [16].

However, despite all benefits and efforts, adopting home gardens is still challenging in Tuvalu because low soil fertility warrants labour-intense composting. Under the background of global warming, frequent sea-water flooding increases salinity in the coral sand in Tuvalu and necessitates the use of off-the-ground cultivation racks [28,29]. Furthermore, it requires individualised strategies considering population density, rainfall, farmland accessibility, among others to optimise this local production network of nutritious food [30,31]. Our results suggest home garden intervention in the outlying islands, especially the Southern islands, could be a solution to establishing an in-country food supply chain, decreasing transportation and preservation costs for Tuvaluans, and reducing carbon footprints from international shipment to indirectly mitigate the long-term impact of global warming [12,32]. In this model, home gardens benefit at the individual, national as well as global levels. Traditionally, people in Tuvalu, like other Pacific islanders, relied on their agroforestry and fishery resources to create sustainable systems which met their dietary needs throughout their lives [10]. Compared to Funafuti where there are much higher levels of urbanisation, people in the outlying islands preserve most of the original lifestyles, and the additional benefits of home gardening under such circumstances may thus become less prominent. Nevertheless, home gardening on Funafuti is still crucial and encouraged due to the additional behavioural modifications to try to decrease obesity and severe obesity that were not seen on the outlying islands. Considering the limited household space for home garden setup, whether occasional participation in centralised government gardens confers similar benefits, albeit to a lesser degree, would be interesting to know for urban planning. These findings can be partially understood in the context of urbanisation.

There are several strengths of this study. First, our study provided first-hand and best available data of home garden use and health outcomes in Tuvalu during the COVID-19 pandemic. Second, our data are of high quality, generalisability, and representativeness, covering more than 10% of the population in the whole nation and collected by trained personnel in a standardised approach. Lastly, we established a sustainable and scalable structure which has laid the foundation for independent local interviewers to collaborate in future projects. The limitations include causality inference given the cross-sectional nature, recall and social desirability response bias inheritance to interview-type questionnaires [33] and unadjusted confounding factors. Convenient sampling is used due to the challenges in implementing randomisation sampling, so participants in this study may not fully represent all Tuvaluan adults. In addition, our result reflects the association between having a home garden and decreased odds for severe obesity, but it is important to note that our study does not account for actual utilisation or specific details, such as the size of the home garden or the duration of gardening activities.

In conclusion, this study identified a clear association between home gardening and obesity in Funafuti, Tuvalu's main island. Although there is limited literature discussing the association of home gardening and health outcomes in Tuvalu or South Pacific countries specifically, randomised trials in the US have shown that investing in home gardens appears to be a promising strategy for promoting fruit and vegetable intake [34,35]. Nevertheless, it is crucial to acknowledge the distinctive ecosystem conditions in each country. When cultivating localised food systems, promoting sustainable gardens, and decreasing imported food reliance, the ecosystem contexts need to be considered, especially in densely populated regions such as Funafuti. We envision home gardens as a supplement to provide affordable products and a vessel to promote health behaviours. Further studies are warranted to disentangle the associations between farming behaviours and health outcomes, including obesity and other metabolic diseases, to address the nutrition-related public health crisis.

## CONCLUSIONS

Having a home garden is associated with lower odds of severe obesity in Tuvalu, and the association is stronger in Funafuti. Smokers are less likely to have home gardens, and increased age is positively associated with having home gardens. These findings promote more home garden utilisation and provide evidence for targeted interventions in Tuvalu.

## Additional material


Online Supplementary Document
