# Supplementary material for: Exploring the link between home garden use and severe obesity: Insights from a nationwide survey in Tuvalu
Source: J Glob Health. 2023 Sep 1;13:04097. doi: 10.7189/jogh.13.04097 (PMC10472202; doi:10.7189/jogh.13.04097)
Supplement: Online Supplementary Document [file jogh-13-04097-s001.pdf]

Supplement Table 1. *Number of participants from each outlying island in the study.*

Island name		Count (%)
Main island (Funafuti)		630 (61.5)
North islands		
	Nanumea	50 (4.9)
	Nanumaga	49 (4.8)
	Niutao	50 (4.9)
Middle islands		
	Vaitupu	76 (7.4)
	Nui	60 (5.9)
	Nukufetau	59 (5.8)
South islands		
	Nukulaelae	30 (2.9)
	Niulakita	20 (2.1)

a. Outlying islands are grouped into three island groups: (1) North three islands (including Nanumea, Nanumaga, and Niutao), (2) Middle three islands (including Vaitupu, Nukufetau, and Nui), and (3) South two islands (including Nukulaelae and Niulakita).

Supplement Table 2. *Multivariable regressions between home garden use and obesity prevalence, among population without non-communicable diseases* [a]

Predictor variables	Unadjusted model	Fully adjusted model [b]	Overlap weight model [c]
**(a) Obesity**	Odds ratio (95% confidence interval and p-value)		
Home garden use	0.914 (0.657-1.279, p=0.596)	0.863 (0.611-1.226, p=0.409)	0.967 (0.904-1.036, p=0.343)
**(b) Severe obesity**			
Home garden use	0.771 (0.515-1.135, p=0.196)	0.738 (0.488-1.099, p=0.142)	0.955 (0.901-1.012, p=0.119)

a. Obesity was defined as BMI >30 kg/m^2, and severe obesity as BMI >40 kg/m^2.

b. Adjusted for gender (male or female), age (grouped in ten years), education level (high school or above), income (>200 AUD or not), and smoking. Frequent consumers are people with response as "almost daily" or "several times a week" for each food item.

c. Weighted for gender (male or female), age (grouped in ten years), education level (high school or above), income (>200 AUD or not), and smoking.

Abbreviation: AUD, Australian dollar; BMI, body mass index; NCD, non-communicable disease.

Supplement Table 3. *Multivariable regressions between home garden use and obesity prevalence, among population without non-communicable diseases in Funafuti* [a]

Predictor variables	Unadjusted model	Fully adjusted model [b]	Overlap weight model [c]
**(a) Obesity**	Odds ratio (95% confidence interval and p-value)		
Home garden use	0.835 (0.551-1.277, p=0.400)	0.779 (0.502-1.217, p=0.268)	0.946 (0.862-1.038, p=0.240)
**(b) Severe obesity**			
Home garden use	**0.552 (0.305-0.951, p=0.039)**	**0.523 (0.284-0.916, p=0.029)**	**0.919 (0.856-0.986, p=0.019)**

a. Obesity was defined as BMI >30 kg/m^2, and severe obesity as BMI >40 kg/m^2.

b. Adjusted for gender (male or female), age (grouped in ten years), education level (high school or above), income (>200 AUD or not), and smoking. Frequent consumers are people with response as "almost daily" or "several times a week" for each food item.

c. Weighted for gender (male or female), age (grouped in ten years), education level (high school or above), income (>200 AUD or not), and smoking.

Abbreviation: AUD, Australian dollar; BMI, body mass index; NCD, non-communicable disease.
